# Avoidant Personality Disorder versus Social Phobia: The Significance of Childhood Neglect

**DOI:** 10.1371/journal.pone.0122846

**Published:** 2015-03-27

**Authors:** Ingeborg Eikenaes, Jens Egeland, Benjamin Hummelen, Theresa Wilberg

**Affiliations:** 1 Department of Group Psychotherapy, Division of Mental Health and Addiction, Vestfold Hospital Trust, Tønsberg, Norway; 2 Department of Research, Division of Mental Health and Addiction, Vestfold Hospital Trust, Tønsberg, Norway; 3 Department of Research and Development, Division of Mental Health and Addiction, Oslo University Hospital, Oslo, Norway; 4 Department for Research and Development, Division of Mental Health and Addiction, Oslo University Hospital, Oslo, Norway; University of New South Wales, AUSTRALIA

## Abstract

**Objectives:**

Avoidant personality disorder (AvPD) and social phobia (SP) are common disorders both in the community and in clinical settings. Whether the two disorders represent different severity levels of social anxiety disorder is currently in dispute. The relationship between AvPD and SP is probably more complex than previously assumed. Several environmental, temperamental, and constitutional factors may play a role in the etiology of AvPD and SP. Better knowledge about childhood experiences may shed light on similarities and differences between the two disorders. The aim of this study was to compare self-reported childhood experiences in AvPD and SP patients.

**Design:**

This is a cross-sectional multi-site study of 91 adult patients with AvPD and/ or SP. We compared patients with AvPD with and without SP (AvPD group) to patients with SP without AvPD (SP group).

**Methods:**

The patients were examined using structured diagnostic interviews and self-report measures, including Child Trauma Questionnaire, Parental Bonding Instrument, and Adult Temperament Questionnaire.

**Results:**

Both AvPD and SP were associated with negative childhood experiences. AvPD patients reported more severe childhood neglect than patients with SP, most pronounced for physical neglect. The difference between the disorders in neglect remained significant after controlling for temperamental factors and concurrent abuse.

**Conclusions:**

The study indicates that childhood neglect is a risk factor for AvPD and may be one contributing factor to phenomenological differences between AvPD and SP.

## Introduction

Avoidant personality disorder (AvPD) and Social phobia (SP) are common disorders both in the general population and in clinical settings [1–5]. There is an ongoing debate regarding whether AvPD and SP are different disorders, or just reflect different degrees of severities of social anxiety disorder (SAD) [6–10]. The introduction of the diagnostic specifier generalized SP (GSP) in the revised third edition of the Diagnostic and Statistical Manual for Mental Disorders (DSM-III-R) [11], defining GSP as fears in most social situations, has brought the diagnostic constructs even closer together. A vulnerable temperament combined with early environmental risk factors are suggested etiological factors in the development of both AvPD and SP [12–14]. In a large study of female twins Reichborn-Kjennerud et al. [15] found that AvPD and SP were influenced by the same genetic factors, while the environmental factors influencing the two disorders were uncorrelated and unique to each disorder. So far, however, our knowledge of environmental influences associated with the two disorders is sparse. We wanted to compare self-reported childhood experiences for patients with AvPD and SP to shed further light on the relationship between the two disorders.

In the DSM-IV [16] AvPD is defined as a pervasive pattern across time and situations, starting in early adulthood, and characterized by social inhibition, feeling of inadequacy and hypersensitivity to negative evaluation, indicated by at least four of seven explicit criteria (Table 1). Social Phobia is defined as a marked and enduring anxiety for one or more social situations in which the person is exposed to or observed by unknown people. The person fears to do something or behave in a way that will be humiliating or embarrassing, and avoids the situations, or endures the situations with intense anxiety or distress. AvPD has primarily been studied in samples of SP [10]. These studies have documented a quantitative severity continuum with an increasing gradient of symptoms and psychosocial dysfunctioning from simple SP, via GSP to GSP with AvPD. Based on these findings a continuum hypothesis has been proposed, suggesting that SP and AvPD represent different conceptualizations of the same disorder, merely differing in degree of severity [10]. Only few studies have included a “pure” AvPD group without SP [1, 2, 17]. The discussion whether AvPD and SP is the same disorder, is part of a larger debate of the relationship between clinical disorders on Axis I and PDs on Axis II, see Kreuger, 2005 [18] and Dimaggio et al 2013 [19]. The discussion parallels the debate on schizophrenia-like symptoms, affective instability, impulsivity and depressive symptoms across Axis I and II, for instance the proposal to categorize Borderline PD as a bipolar spectrum disorder [20, 21].

**Table 1:** DSM-IV diagnostic criteria for AvPD.

1.	Avoids occupational activities that involve significant interpersonal contact because of fears of criticism, disapproval, or rejection
2.	Is unwilling to get involved with people unless certain of being liked
3.	Shows restraint within intimate relationships because of the fear of being shamed or ridiculed
4.	Is preoccupied with being criticized or rejected in social situations
5.	Is inhibited in new interpersonal situations because of feelings of inadequacy
6.	Views self as socially inept, personally unappealing, or inferior to others
7.	Is unusually reluctant to take personal risks or engage in any new activities because they may prove embarrassing

In a review of environmental risk factors for SAD, Brook & Schmidt [12] found studies of four areas: parenting and family environment, adverse life events, socioeconomic status and culture, and gender. The authors conclude that “research has successfully correlated parenting as a small but integral part of the mechanism in developing SAD”, and points to an interrelated multi-faceted process of environmental risk and resilience factors in development of the disorder [12]. More recently in the study of Kuo, Goldin, Werner, Heimberg, & Gross [22] individuals with GSP reported greater childhood emotional abuse and neglect, but not more sexual abuse, physical abuse, or physical neglect, compared with healthy controls, pointing to the less dramatic and more subtle maltreatment as a possible risk factor in the development of GSP. Like for most studies of SP or SAD, co-morbidity with AvPD was not controlled for. Moreover, like most studies on childhood trauma, the relative contribution of neglect and abuse was not investigated. We wanted to elaborate on these findings, by including AvPD and examine the relative contribution of abuse and neglect, and even temperament.

Childhood trauma and parental maltreatment are also well documented as risk factors for adult PD in general, in both prospective and retrospective studies [23–26]. Johnsen et al. [24] found that individuals with documented childhood abuse or neglect were four times as likely as those who were not abused or neglected, to be diagnosed with PDs during early adulthood. Childhood emotional neglect was associated with increased risk of several PDs, including AvPD [27].

So far, few studies have focused specifically on AvPD and childhood experiences. However, in a large clinical study Rettew et al. [28] found that patients with AvPD reported more physical and emotional abuse during childhood compared to patients with major depression, but this result was influenced by comorbid diagnoses. In a large outpatient sample in Shanghai, self-reported experience of childhood emotional neglect was associated with adult cluster C PDs (avoidant, dependent, and obsessive-compulsive PD) [26]. Joyce et al. [13] found that self-reported childhood neglect predicted AvPD in a sample of depressed outpatients. Moreover, in an early, small retrospective study, Arbel and Stravynski [29] found that the main features differentiating adult AvPD patients from healthy controls were the perception of a discouraging home climate with less parental demonstration of love and pride in the child, and a perception of their parents as shaming, guilt-engendering, and intolerant.

Abuse refers to maltreatment, harmful behavior, and non-accidental injury from an adult person directed toward the child, while neglect refers to the failure of caretakers to provide a child’s basic psychological or physical needs [30]. Generally, childhood neglect has received less empirical attention than childhood abuse [31]. In clinical settings the experience of neglect in childhood may be overshadowed by dramatic histories of maltreatment and abuse. However, parental abuse and neglect can co-occur in dysfunctional families, making it difficult to disentangle specific consequences of the various types of maltreatment that the child may suffer [32]. At present little is known about the unique contribution of neglect to adult psychopathology more generally, and to AvPD as compared with SP specifically.

Parental behavior has also been studied with the Parental Bonding Instrument (PBI) [33], which aims to collect relevant retrospective information of childhood experiences. A combination of low scores on the two subscales *care* and *control* is called the *neglectful parenting* pattern, which was found to be the dominating pattern in a small sample of patients with AvPD [34]. However, when Joyce et al. [13] found neglect to be associated with AvPD, they operationalized neglect as low scores on the care dimension only. A combination of low scores on the care and high scores on the control subscales is called the *cold control* pattern, which has been associated with many kinds of adult psychopathology [35–37]. The cold control pattern might also be among the risk factors for SAD [12, 38, 39].

Maltreatment probably interacts with temperamental factors to influence personality development and risk of psychiatric symptoms [13]. Rothbart and Derryberry [40] define temperament as constitutionally based individual differences in emotional, motor, and attentional reactivity and regulation. Temperament is influenced by experience, and in turn influences experience, and is gradually transformed and integrated into our adult personality [41]. Shyness has been proposed as a temperamental trait in both SAD and AvPD. However, Prior, Smart, Sanson, and Oberklaid [42] found only modest relation between childhood shyness and adolescent anxiety disorder in a longitudinal, community study. Most shy children did not develop an anxiety disorder, and most adolescents with anxiety disorders had not been especially shy. Nevertheless, other studies of both epidemiological and clinical samples suggest that both AvPD and SP are associated with the temperamental factor “behavioral inhibition”, which is characterized by avoidance of strangers and novelty, shyness, heightened sensitivity and anxiety reactivity [43–45]. Thus, these disorders seem to have some temperamental dispositions in common, but temperamental manifestations could still be present in various degrees.

Taken together, some studies indicate that both AvPD and SP are associated with various types of childhood maltreatment. Most notable, however, no studies have made a direct comparison of childhood environmental factors between the disorders.

In clinical samples, many patients with AvPD have a co-occurring SP diagnosis [10]. Also in this sample the two diagnoses were concurrent in most patients. As we wanted to focus on AvPD, we divided the patients into two diagnostic groups: patients with AvPD with or without SP (the AvPD group) and patients with SP without AvPD (the SP group). This choice was supported by previous findings in a large clinical PD sample [17], partially by the results of an epidemiological study [1], as well as a previous study of the present sample [7] suggesting that SP in subjects with AvPD does not add to the overall severity of the condition.

The aim of the present study was to investigate similarities and differences between the two diagnostics groups in self-reported childhood experiences. Based on previous research we expected that 1) Patients in the AvPD group will report more severe childhood maltreatment than patients in the SP group as assessed by CTQ. 2) We further explored the relationship between the diagnostic groups and childhood maltreatment when taking into account the presence of different trauma. 3) Moreover, we expected that the AvPD group will more often report a neglectful parenting style compared with the SP group, measured by PBI, whereas the groups will not differ in rates of a cold control parenting style. 4) Finally, we hypothesized that differences in environmental factors will remain significant when controlling for temperament.

## Methods

### Settings

This cross-sectional multi-site study was conducted by the Vestfold Hospital Trust and included 91 adult patients with AvPD and/or SP. Exclusion criteria were cluster A or B PDs, current alcohol or substance dependence, psychotic disorders, bipolar I disorder, adult attention deficit hyperactivity disorder (ADHD), pervasive development disorders (e.g., Asperger’s syndrome), organic syndromes, and homelessness. Twenty-five of the included patients were selected using baseline data from the Ullevål Personality Project (UPP) [46]. The other 66 patients were recruited by their therapists, regardless of time in therapy, from seven treatment centers specialized in treating PDs or anxiety disorders. The recruitment of these patients was based on the therapist’s diagnostic screening and evaluation, and inclusion was decided after the diagnostic research interviews. Seventy-two patients were recruited. Six patients were excluded: five because the research interviews revealed diagnoses of alcohol dependence (*n* = 2), adult ADHD (*n* = 2), and borderline PD (*n* = 1); and one patient dropped out before completing the interviews. Study participation was voluntary, and all patients provided informed written consent before inclusion. The study was approved by the Norwegian Social Science Data Service and the Regional Committee for Medical Research Ethics.

### Assessments

#### Axis I and axis II diagnoses

Axis I diagnoses were based on the Mini International Neuropsychiatric Interview for Axis I diagnoses (MINI) [47]. The Structured Clinical Interview for the 4^th^ edition of the Diagnostic and Statistical Manual of Mental Disorders (DSM-IV) was used to assess personality disorders (SCID-II) [48]. Trained and experienced clinicians conducted the interviews. All patients with SP were asked to describe examples of SP-related problems from their lives, which were used along with other information to evaluate whether they had simple or generalized SP. All interviews were audiotaped. An independent, blind, and experienced psychiatrist rated the diagnostic interviews of 26 randomly selected patients. Although there was 85% agreement regarding the presence or absence of SP, there were too few patients without SP to compute Kappa for SP. Kappa for AVPD was 0.76. The intraclass correlation (ICC 2.1) was 0.86 (95% CI: 0.71–0.93) for the number of avoidant criteria and 0.89 (95% CI: 0.75–0.95) for the total number of PD criteria, indicating satisfactory diagnostic reliability.

#### Childhood trauma

Child trauma was assessed using the Childhood Trauma Questionnaire (CTQ) a 44-item self-report inventory that provides brief screening for histories of abuse and neglect, and has shown good reliability and validity [49, 50]. Items are scored on a 5-point Likert scale from 1 (never true) to 5 (very often true). Items scored are summed on 5 different subscales: emotional, physical, and sexual abuse; and physical and emotional neglect. *Emotional abuse* refers to verbal assaults on a child’s sense of worth or well-being, or any humiliating, demeaning, or threatening behavior. *Physical abuse* includes descriptions of bodily assaults that pose a risk of or result in injury. *Sexual abuse* describes sexual contact or conduct. *Emotional neglect* refers to lack of love, encouragement, belonging, and support. *Physical neglect* refers to lack of food, shelter, safety, supervision, and health. It is not unusual to underreport childhood trauma [51] and CTQ therefore includes a 3-item denial/minimization scale to detect false negative trauma reports. In this sample, eight patients scored on the denial scale. Still, no differences were detected when data were analyzed with and without these eight patients. The raw scores of the five CTQ subscales are not comparable and have different cut-off thresholds. Raw scores were therefore converted into classifications of four levels of severity based on validation studies in normal and psychopathological samples [30]: “absent to minimal” (level 1), “low to moderate” (level 2), “moderate to severe” (level 3), and “severe to extreme” (level 4). We used these four levels of severity to harmonize the subscales. Thereby we were able to compute a composite neglect score based on the average classification of severity (1–4) of emotional and physical neglect. Likewise, we computed a composite abuse score from the three abuse subscales: emotional abuse, physical abuse and sexual abuse. The correlation between physical and emotional neglect was not significant. Emotional abuse correlated moderately with both physical (*r* = .443) and sexual abuse (*r* = .372) whereas the correlation between sexual and physical abuse was non-significant. This led us to conclude that colinerality did not prohibit the construction of the composite scores.

#### Perceived parental behavior

**Fig 1:**
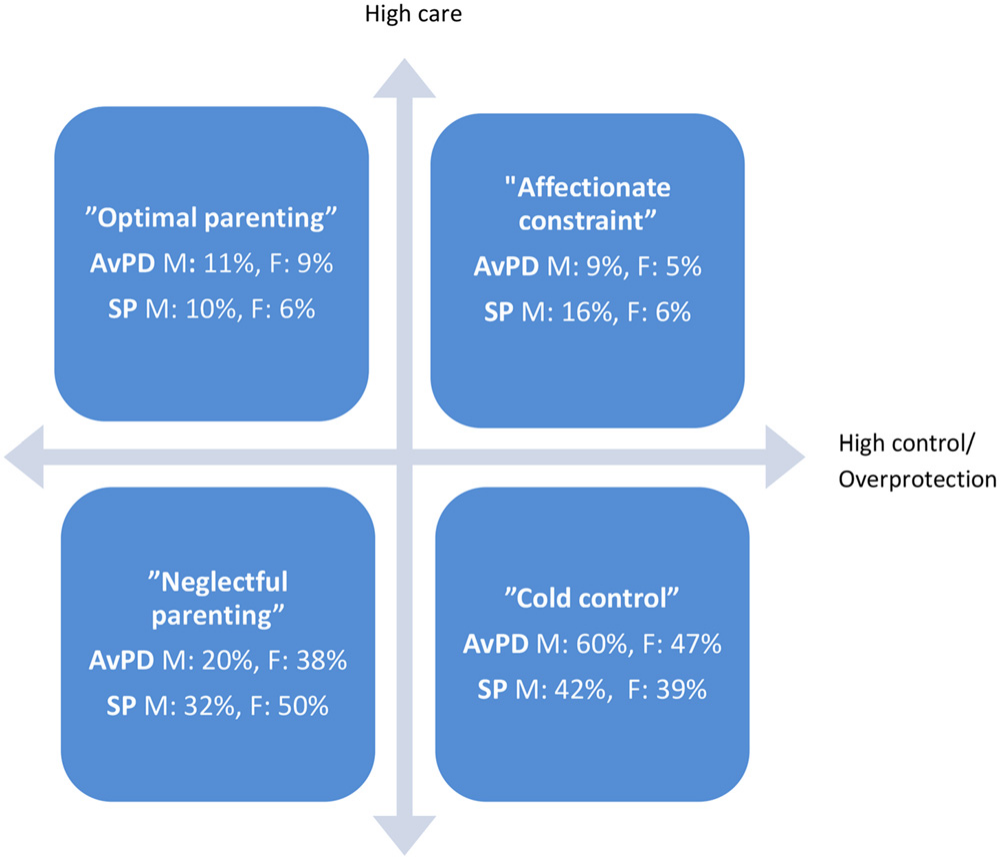
Attachment style in Avoidant personality disorder (AvPD, n = 70) and Social phobia (SP, n = 20). Distribution of the AvPD and SP groups in the two dimensional, four categorical parental style model according to the Parental Bonding Instrument (PBI). ORIGO is defined as the cut-off scores, se text. AvPD: Avoidant personality disorder, SP: Social phobia, M: Mother, F: Father

Perceived parental behavior was assessed by the 25-item Parental Bonding Instrument (PBI) self-report questionnaire [52]. The items are scored on a 5-point Likert scale from 1 (very likely) to 5 (very unlikely), based on perceived parental behavior before the age of 16 years. Usually two subscales are computed for each parental figure: care/affection and overprotection/control. Combination of the two subscales generates four parental styles: *optimal bonding* (high care, low overprotection); *affectionate constraint* (high care, high overprotection); *affectionless control*, also called *cold control* (low care, high overprotection); and *neglectful parenting* (low care, low overprotection). Cut-off scores for high and low *care* and *overprotection* were computed based on normative data [33]. The respective cut-off scores are 27 and 24 for maternal and paternal *care*, and 13.5 and 12.5 for maternal and paternal *control*. We used these cut-offs to compute the four patterns (Fig. 1).

#### Temperament

The short form of the Adult Temperament Questionnaire (ATQ) [41] was used to assess temperament. The ATQ is a self-report questionnaire that consists of 77 items rated on a 7-point Likert scale and includes four factor scales: effortful control, negative affect, extraversion, and orienting sensitivity. Good internal consistency was reported in a sample of 258 undergraduate students, with Cronbach’s alpha of 0.78, 0.81, 0.75, and 0.85 for effortful control, negative affect, extraversion, and orienting sensitivity, respectively [53]. The same level of internal consistency was observed for the Norwegian version of the ATQ in the UPP sample, with Cronbach’s alphas of 0.82 for effortful control, 0.81 for negative affect, 0.73 for extraversion, and 0.81 for orienting sensitivity [Urnes et al., unpublished data].

### Participants

The 91 patients had a mean age of 37.6 years (*SD* = 10.2); 65% were female, 44% were married or cohabiting, and they had an average of four years of education after junior secondary school (*SD* = 3.2). The mean age at first contact with psychiatric services was 26.9 years (*SD* = 10.1), and the average time interval between the first contact and the present treatment was 12 years. The sample was divided into two groups, the AvPD group (AvPD with and without SP; *n* = 71) and the “pure” SP group (SP without AvPD; *n* = 20). Eighteen patients in the SP group (90%) had generalized SP, two had simple SP. There were no differences in socio-demographic characteristics between the two groups, except that only 29% of patients in the AvPD group were working half-time or more, compared with 60% in the SP group (*p =* 0.011; Table 2). The AvPD group also had more Axis I or “symptom disorders” (*p =* 0.029) and a greater number of total PD criteria than the SP group (*p <* 0.001). More details are provided in a previous publication [7].

**Table 2:** Socio-demographic and clinical characteristics of patients with AvPD^a^ (n = 71) and SP^b^ (n = 20).

	AvPD	SP	t(df) or χ2(df)	p
Female, %	69	50	2.47(1)	0.116
Age, years	37.4 (9.4)	38.4 (12.6)	0.33 (25)	0.743
Married/Cohabiting, %	40	50	0.64 (1)	0.452
Education after primary school, years	3.9 (3.3)	4.4 (3.0)	0.53 (88)	0.601
Age at first contact psych. services, years	26.9 (10.4)	26.7 (9.1)	0.11 (88)	0.910
Quality of life	3.9 (1.5)	3.9 (1.4)	0.15 (89)	0.883
50–100% Occupation, %	29	60	6.47 (1)	0.011
Number of symptom disorders	3.1 (1.12)	2.5 (.89)	2.22 (89)	0.029
Total number of PD criteria	12.5 (3.99)	6.8 (2.69)	7.55 (45.2)	0.000
Work and social adjustment score	24.2 (69)	19.1 (9.3)	2.68 (88)	0.009

Data are presented as mean (SD) unless otherwise noted.

^a^ The AvPD group comprised patients with AvPD with and without co-occurring SP.

^b^ The SP group comprised patients with SP without AvPD.

### Statistics

Background demographic and clinical variables were analyzed using *t*-tests and the chi-squared test for continuous and categorical variables, respectively. We compared the AvPD group and the SP group, applying analysis of variance (ANOVA) for the continuous variables of the CTQ, ATQ, and PBI. Because skewed distributions of the separate abuse subscales of the CTQ precluded the use of parametric methods, the Mann-Whitney-U test was used. To analyze the unique contribution of the variables showing significant group differences in the ANOVAs, follow-up covariance analyses (ANCOVAs) were performed controlling for the effects of these possible confounding variables. We used *Eta^2^* and *r^2^* as measures of effect size for the normal and the skewed distributed data respectively. The alpha level was set at *p* < 0.05. Some statistically trends are reported due to the small sample size and risk of type II errors.

## Results

In line with our first hypothesis, the AvPD group had significantly higher scores on physical neglect, as well as on the composite neglect variable, compared with the SP group (*p* = 0.003 and *p* = 0.007), Table 3. Emotional neglect trended higher in the AvPD group (*p* = 0.061). However, contrary to our expectations, there were no significant differences in emotional, physical, or sexual abuse between the groups although there was a trend toward more abuse in the AvPD group, measured by the more robust composite abuse variable (*p* = 0.069), Table 3.

**Table 3:** Clinical Characteristics of Patients with AvPD^a^ (*n* = 71) and SP^b^ (*n* = 20).

		AvPD	SP	*P*	Effect size
		Mean (SD)/ Mdn (I-R)^*e*^	Mean (SD)/ Mdn (I-R)^*e*^		*Eta^2^/ r^2^* ^*f*^
**CTQ** ^***c***^					
	Emotional abuse	11.00 (8) ^***e***^	10.00 (6) ^***e***^	.144	.023 ^*f*^
	Physical abuse	5.00 (1) ^***e***^	5.00 (1) ^***e***^	.668	.002 ^*f*^
	Sexual abuse	6.00 (4) ^***e***^	5.50 (2) ^***e***^	.354	.009 ^*f*^
	Composite Abuse Class ^***d***^	1.9 (0.8)	1.6 (0.6)	.069	.037
	Emotional neglect	16.0 (4.2)	13.7 (5.2)	.061	.039
	Physical neglect	8.3 (3.2)	6.7 (1.6)	.003	.050
	Composite Neglect Class ^***d***^	2.8 (0.7)	2.3 (0.7)	.007	.081
**PBI**					
	Mother care	17.7 (9.5)	19.8 (7.7)	.363	.010
	Mother control	17.4 (7.8)	17.6 (8.7)	.899	.000
	Father care	14.9 (8.2)	16.2 (7.7)	.568	.004
	Father control	14.5 (7.4)	14.1 (5.9)	.807	.001
**ATQ**					
	Negative Affect	4.9 (0.7)	4.4 (0.8)	.010	.072
	Extraversion	3.2 (0.8)	3.6 (0.9)	.078	.034
	Effortful Control	4.1 (0.8)	4.3 (0.7)	.259	.014
	Orienting sensitivity	4.3 (0.8)	4.2 (0.7)	.484	.006

^a^ The AvPD group comprised patients with AvPD with and without co-occurring SP.

^b^The SP group comprised patients with SP without AvPD.

^*c*^Score range: 5 (no) to 25 (extreme),

^*d*^Score range: 1 (none or minimal) to 4 (severe to extreme)

^*e*^Mdn = Median; I-R = Interquartile Range,

^*f*^ *r^2^* = (z /√N) *^2^*

Exploration of the relative contribution of neglect and abuse revealed that the AvPD-related difference in the composite neglect remained significant when controlling for physical abuse (*F* = 6.33, *p* = 0.014, *Eta* *^2^* = 0.068), sexual abuse (*F* = 6.63, *p* = 0.012, *Eta* *^2^* = 0.071), and emotional abuse (*F* = 5.61, *p* = 0.020, *Eta* *^2^* = 0.061) by ANCOVA (*df*: 2, 91). Each of the abuse variables was also significantly related to neglect: physical abuse (*F* = 9.20, *p* = 0.003, *Eta* *^2^* = 0.096); sexual abuse (*F* = 5.56, *p* = 0.021, *Eta* *^2^* = 0.060); and emotional abuse (*F* = 29.45, *p* = 0.000, *Eta* *^2^* = 0.25).

Contrary to our hypothesis, there were no between-group differences in the distribution of the four categories of parenting patterns of the PBI when analyzed by a Chi Square test of the 4 x 2 table (Fig. 1). Neither were there any differences in the dimensional scales *care* nor *control* between the groups. The great majority in both groups reported their parents to be low on *care* (80% of mothers and 86% of fathers in the AvPD group, and 74% of mothers and 89% of fathers in the SP group).

Scores on the ATQ factor *negative affect* were higher *(p* = 0.010) among patients in the AvPD group, compared with the SP group and *extroversion* trended lower *(p* = 0.078) in the AvPD group, Table 3. To determine whether difference in the composite neglect was significant when controlling for these differences in temperament, we used the composite neglect score as dependent variable: In agreement with the last hypothesis, the AvPD-related difference in neglect remained significant when we controlled for the temperamental factors by ANCOVA *(df*: 2, 91; controlling for *extroversion*: *F* = 6.99, *p* = 0.010, *Eta* *^2^* = 0.074; controlling for *negative affect*: *F* = 5.27, *p* = 0.024, *Eta* *^2^* = 0.057). Neither *extroversion* nor *negative affect* was significantly related covariates of the composite neglect score.

## Discussion

The main finding of the present study was that AvPD was associated with more self-reported neglect as compared with SP. This result is partially in agreement with retrospective findings that emotional neglect was predictive of cluster C PDs [26] and prospective findings that it was predictive of AvPD [27]. The difference between the diagnostic groups was most pronounced for physical neglect, though. Notably, the AvPD-related difference in neglect remained significant when we controlled for child abuse as well as temperamental differences. Thus, the results suggest that the often less dramatic or silent maltreatment experiences of emotional and physical neglect might be risk factors for adult AvPD over and above the effects of both abuse experiences and temperamental dispositions.

Neglect is generally defined as the absence of protection, care, and positive attention (i.e., the parents are physically present, but do not attend to the child). No doubt, neglect is potentially a very harmful experience for a child and the psychological consequences are probably manifold. From the child’s perspective, his or her physical and emotional needs may be perceived as irrelevant or too much trouble for the parents. It is easy to understand that a child who is not given attention, care, and protection within the attachment relationship might develop assumptions that one is not of interest to others or not worthy of being loved. Correspondingly, neglect may interfere with the child’s development of perceptions of others as safe sources of comfort, support, and reassurance. Such experiences could be integrated in the personality as more permanently disturbed representations of self and others characteristic for this form of personality pathology. In instances of more severe neglect even basic affect regulation and self-coherence may be affected. Moreover, individuals with a negative self-image and lack of interpersonal trust tend to relate to others in maladaptive ways. For example, they may not ask for others opinions or involvement regarding their personal issues, because of expectations of others as not interested or critical to their needs. Also, close relationships may evoke inherent negative self-views causing defensive avoidance of social contact. Others may interpret this behavior as expressing no need or wish for their involvement, or even as arrogant, giving rise to even more interpersonal distance. Thus this interpersonal pattern may reinforce itself in a vicious circle confirming their assumptions and increasing their loneliness.

The described patterns are in line with the self-other pattern described in the DSM-5 regarding AvPD [54]. According to the alternative model for PDs in DSM-5, Section III, a defective self as well as relational dysfunction is at the core of PDs [54, 55]. In a previous study of the present sample, patients with AvPD exhibited more impairment in self and relational functioning than those with SP [7]. One hypothesis, then, is that different levels of childhood maltreatment is one of the factors underlying such differences in personality functioning. In the presence of common constitutional dispositions for AvPD and SP [15], the experience of more severe childhood neglect may be a factor that drives personality development towards avoidant personality pathology [13]. It is important to recognize, however, that a child’s temperamental make-up may have a bidirectional effect, i.e. exert a certain influence on the parents’ child-rearing behavior, and at the same time render the child more vulnerable to specific responses from the caregivers. Maltreatment probably affects children differently, and interacts with both constitutional and environmental risk and resilience factors. For example, temperamental factors may render a person more vulnerable to developing AvPD or SP if they are exposed to neglect or abuse, and also more at risk for being neglected or abused. Thus, the pathways to specific disorders are complex and more studies are needed to increase our knowledge of the relationship between childhood experiences, temperament, and personality dysfunctioning in subjects with AvPD and SP.

The severity level of the mean score for *emotional neglect* was in the moderate-to-severe range in the AvPD group and in the low-to-moderate range in the SP group. For *physical neglect*, the severity level was in the low-to-moderate range in the AvPD group and in the none-to-minimal range in the SP group. Interestingly, based on visual comparison of our data with a Spanish sample in which the CTQ was applied to patients with borderline PD (BPD), other PDs, and non-psychotic axis I disorders [23], our AvPD group seems to have higher neglect scores than the BPD group, and our SP group resembles the same level as the BPD group. Compared with a sample of incarcerated boys in the Netherlands also using CTQ [32], our groups seems to exhibit more severe scores on neglect and emotional abuse, similar scores on sexual abuse, and somewhat milder scores on physical abuse. Thus, the level of self-reported neglect in our AvPD group was substantial.

In contrast to the results based on the CTQ, no between-group differences were detected regarding the PBI. According to the PBI scores, low *care* and high *control* (i.e., the *cold control* pattern) was frequent in both groups. The fact that the PBI did not differentiate between the disorders in this sample is in agreement with other studies. The dimensions assessed by the PBI might represent non-specific vulnerability factors for psychopathology across various psychiatric disorders [56]. On the other hand, it might also be due to methodological variations. CTQ asks for frequencies of different type of maltreatment, whereas PBI asks how they perceive their parents behavior. The patient’s perception of their parent’s behavior might be influenced or distorted by their actual experiences with the parents.

A main strength of the study was the direct comparison of environmental risk factors in terms of childhood experiences between AvPD and SP. We also took into account temperamental differences between the disorders. Moreover, it is a clinical study with impaired patients with fully developed disorders. The SP group consisted mainly of patients with GSP, potentially making it even harder to detect any differences in comparison with AvPD. However, the results should be interpreted in light of some notable limitations. The small sample size, especially in the SP group, increased the risk of type II errors. Also, few patients with AvPD without co-occurring SP in this sample precluded a comparison of pure diagnostic groups though, a fact that might moderate or camouflage possible differences. Thus, there is a need for replication in larger samples with pure diagnostic groups. The patient sample comprised chronically poorly functioning patients [7]. Excluding patients with cluster A and B PDs further limited the representativeness of the sample. Thus, the results may not be generalized to a broader psychiatric population. Despite these limitations, we detected significant and clinically meaningful differences between the groups. The lack of standardized assessment of simple SP versus GSP (e.g. MINI plus) is another limitation. However, we compensated by systematically asking patients to give examples of the extent of their social anxiety, and then we made a general evaluation of all available information. Further, it should be mentioned that age at the time of maltreatment was not taken into account in this study, i.e., the CTQ asks for the frequency of experiences before 16 years of age. However, the potential damage of childhood neglect and abuse is likely more severe at younger ages and during particular sensitive developmental stages [57, 58].

Self-report of childhood trauma is another major limitation. Retrospective reports of early memories are vulnerable to reconstructive bias resulting from mood and personality, and to the conscious and unconscious processes of “forgetting”. Although certain specific traumatic events may stand out as unusual or extreme, abuse and neglect are often part of an ongoing pattern, and may be stored as scripted or generic memories—reoccurring themes, more than details of the specific events. The CTQ attempts to elicit these scripted memories by asking respondents how often past events happened, rather than the details of traumatic memories [30]. Moreover, underreporting is probably a larger threat to validity in retrospective reports than false positive reports [59]. The denial scale of the CTQ aimed to detect such false negative reports. Few patients in this sample scored on this scale, only two had a full score. In the present sample, patients received different types and length of psychotherapy, which may have influenced their ability to report on childhood trauma. In addition patients with AvPD may have a general difficulty in appreciating mental states [60, 61]. Unconscious processes, such as dissociation and attachment organization, might also lead to an inability to recall traumatic memories [62]. Any differences in attachment organization between AvPD and SP may shed light on their etiology and should be topics for further research.

## Conclusions

To our knowledge this is the first study to compare patients with AvPD and SP regarding childhood experiences, parental behavior, and temperament. Our findings suggest that the experiences of physical and emotional neglect in childhood are risk factors for adult AvPD and SP, most pronounced for AvPD though. The study highlights the potential toll that not being seen, taken care of, and protected during childhood may have on mental health. The relationship between AvPD and SP is complex and further research should aim to recruit larger and pure diagnostic groups. Childhood neglect may be part of the relational histories woven into the identity, self-esteem and interpersonal patterns of patients with AvPD, and may contribute to different degrees of personality dysfunction in patients with AvPD and SP. While in need of more research, this understanding may be useful for the development and facilitation of psychotherapy more specifically tailored to individuals with AvPD.
