# Correction: Avoidant Personality Disorder versus Social Phobia: The Significance of Childhood Neglect

**DOI:** 10.1371/journal.pone.0128737

**Published:** 2015-05-15

**Authors:** Ingeborg Eikenaes, Jens Egeland, Benjamin Hummelen, Theresa Wilberg

The legend for Fig 1, “Attachment style in Avoidant personality disorder (AvPD, n = 70) and Social phobia (SP, n = 20)” is incorrect. The complete, correct Fig 1 legend can be viewed here.

**Fig 1:**
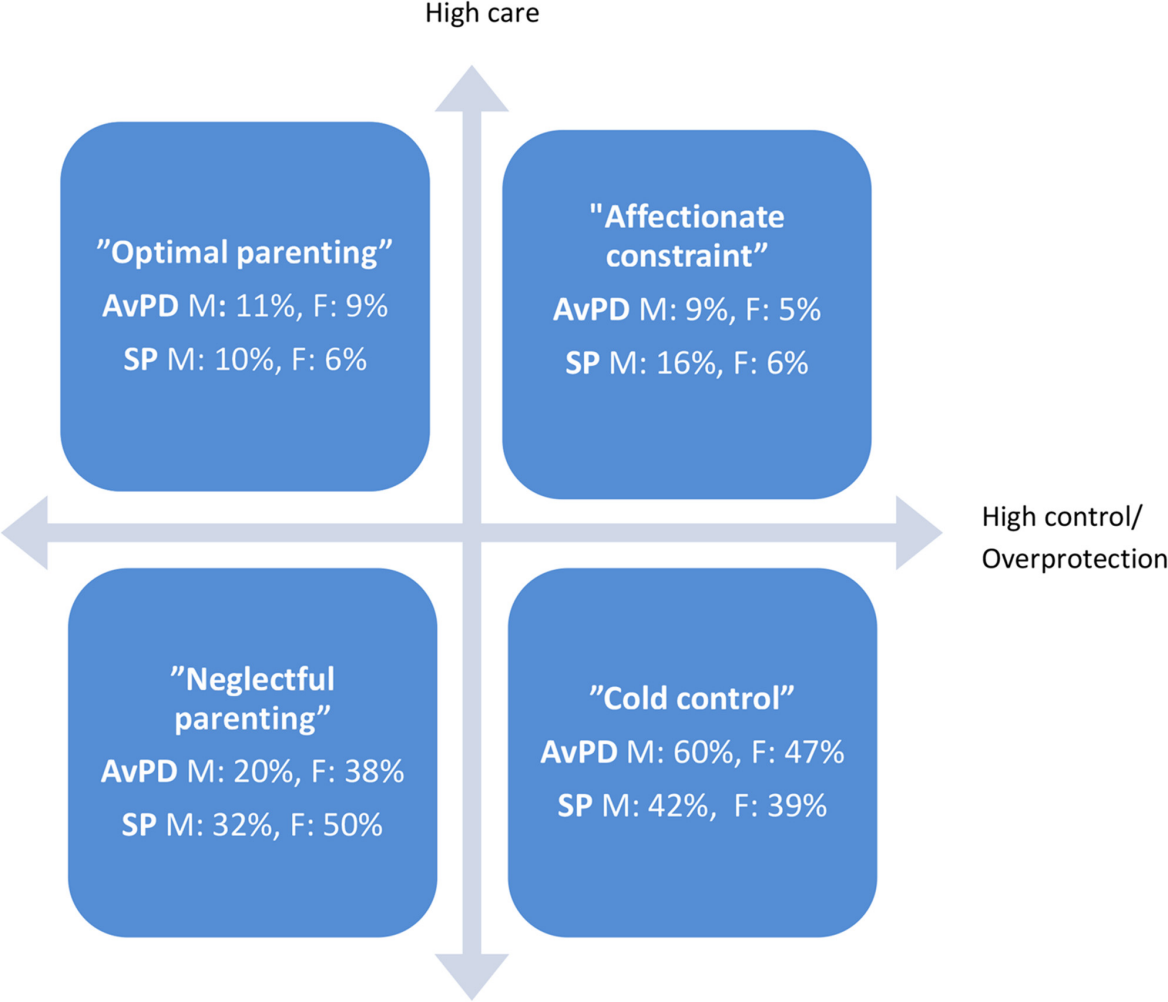
Self-reported parental style in the AvPD and SP groups. Distribution of the AvPD and SP groups in the two dimensional, four categorical parental style model according to the Parental Bonding Instrument (PBI). ORIGO is defined as the cut-off scores, se text. AvPD: Avoidant personality disorder, SP: Social phobia, M: Mother, F: Father
